# Shielding Against Information Overload in the Post-Pandemic Era: The Protective Chain of Family Cohesion, Mindfulness, and Lower Anxiety

**DOI:** 10.3390/bs16020212

**Published:** 2026-01-31

**Authors:** Bingyang Wang, Shangzhe Li, Mengxuan Wu, Jie Wu

**Affiliations:** 1 Key Research Base of Humanities and Social Sciences of the Ministry of Education, Academy of Psychology and Behavior, Tianjin Normal University, Tianjin 300387, China; 2 Faculty of Psychology, Tianjin Normal University, Tianjin 300387, China; 3 Department of Speech, Language and Hearing Science, The University of Texas at Austin, 2504A Whitis Ave. (A1100), Austin, TX 78712-0114, USA; 4 Tianjin Social Science Laboratory of Students’ Mental Development and Learning, Tianjin 300387, China

**Keywords:** information addiction, family cohesion, mindfulness, anxiety, post-pandemic era

## Abstract

Amid the uncertainties of the post-pandemic era, there has been a notable rise in information addiction among individuals, which may function as a coping mechanism in response to perceived situational threats. Family cohesion can function as a protective factor against internet addiction. However, the mechanism by which family cohesion mitigates internet addiction remains largely undiscovered. The study aimed to reveal the role of family cohesion in increasing information addiction behavior and the mediating effects of mindfulness and anxiety in this epidemic. A total of 1043 college students completed an online questionnaire including the Family Adaptability and Cohesion Evaluation Scale (FACESIII), State-Trait Anxiety Inventory (STAI), Mindful Attention Awareness Scale (MAAS), and Information Addiction Scale. (1) Family cohesion and information addiction exhibited a negative correlation; (2) mindfulness and anxiety functioned as mediators within this association; (3) stronger family cohesion was predictive of reduced information addiction behavior through a chain mediating effect, whereby mindfulness negatively predicted anxiety. These findings substantiate the study’s theoretical framework, underscoring the interconnected nature of information addiction during crises.

## 1. Shielding Against Information Overload in the Post-Pandemic Era: The Protective Chain of Family Cohesion, Mindfulness, and Lower Anxiety

In the current post-pandemic context, the lifestyles and social interaction patterns of university students have undergone significant transformations. In recent years, external environmental changes experienced in many regions have compelled individuals to sustain a relatively constrained mode of living over prolonged periods, posing ongoing challenges to public psychological and emotional adaptation (Brooks et al., 2020; Gostin & Wiley, 2020; Pellegrini et al., 2020; Peng et al., 2025).

Environmental uncertainties and the associated sense of constrained resources, such as alterations in daily social interactions and leisure activities, may drive individuals to seek alternative pathways for resource acquisition (Hobfoll et al., 1990). With the reduced accessibility of offline group activities and physical recreational venues, students have correspondingly adapted their classroom learning and extracurricular routines (Pellegrini et al., 2020). The diminished availability of external stimuli has rendered the internet a crucial medium for information acquisition and daily engagement. Particularly during periods of heightened uncertainty, digital platforms such as social media have become pivotal sources of information (Lachlan et al., 2014). In response to environmental shifts, individuals have significantly increased their information-seeking behaviors related to safety, health, and related topics (Spence et al., 2007). Concurrently, excessive reliance on digital networks has led to a growing prevalence of information addiction (Mangono et al., 2021; Bento et al., 2020; Alimoradi et al., 2024), which is associated with a range of psychological and behavioral issues, including diminished quality of life, reduced academic engagement, and emotional distress (Kil et al., 2024; Öztekin, 2024; Chen et al., 2024).

Research indicates that individuals from well-functioning and cohesive families often exhibit greater psychological resilience during stressful environmental transitions (Gouveia et al., 2024; Chai & Shek, 2024). Conversely, strained family relationships may exacerbate an individual’s stress experience (Brik et al., 2024). The emotional support provided by families plays a critical role in helping adolescents navigate the psychological challenges posed by external changes (Trull-Oliva et al., 2024). Notably, individuals from less cohesive families tend to invest more time in online activities, suggesting that robust family functioning may serve as a protective factor against internet-related addictive behaviors among adolescents (Bekada, 2024). Therefore, in the context of the increasingly digitalized lifestyles of contemporary university students, investigating how family cohesion influences information addiction behaviors and clarifying the underlying mediating mechanisms hold significant implications for fostering adolescent mental health and adaptation. 

### 1.1. The Relationship Between Family Cohesion and Information Addiction

Information addiction, which belongs to the generalized concept of internet addiction, is a condition in which individuals compulsively engage in information consumption behavior, characterized by an obsessive drive toward web surfing or information acquisition often propelled by a fear of missing out (K. Young et al., 1999; K. S. Young & Rogers, 1998; Musetti et al., 2016; Z. Zhou & Yang, 2006). Davis’s cognitive-behavioral model (Davis, 2001) offers a theoretical perspective on Pathological Internet Use (PIU). This model underscores that PIU stems from distal causes and proximal causes, suggesting cognitive maladaptation leads to behavioral symptoms. In the Diathesis-Stress framework of distal causes, abnormal behaviors are a combination of intrinsic vulnerabilities (or diathesis) and external pressures. Against the backdrop of significant changes in the external environment, the public’s demand for immediate and accurate information continues to grow, making various digital platforms essential tools for obtaining real-time updates and coordinating daily life. Social applications, represented by WeChat, have become key nodes for information dissemination and public services, thanks to their extensive user base and efficient communication capabilities. Relevant institutions have also collaborated with technology companies to promote the optimization and innovation of information service tools, helping the public adapt to external changes and enhance their resilience. Meanwhile, the positive outcomes of timely access to information, such as helping individuals maintain a sense of control amidst uncertainty and adjust their behavior more effectively to cope with changes further reinforce the sustained behavior of information-seeking (Davis, 2001). 

Furthermore, family cohesion functions as an intrinsic vulnerability of the model. The circumplex model delineates family functions across three primary dimensions: cohesion, adaptability, and communication. Empirical evidence from various family theoretical frameworks and therapeutic methods corroborates the strong interrelationships among these dimensions (Olson et al., 2019). At its core, family cohesion embodies the emotional bonding shared among family members. 

Extensive research illuminates the influence of family cohesion on tendencies towards internet addiction. Individuals who experience lower family functions are more susceptible to internet addiction and more likely to higher internet addiction severity (Yan et al., 2014; Lian et al., 2023; Lochner et al., 2024; Hwang & Silverstein, 2025). Family cohesion negatively predicts internet addiction among adolescents (Sarour & El Keshky, 2023). Considering that association between family cohesion and internet addiction, such as information addiction, we hypothesize that:

**H1.**  *Family cohesion exhibits a negative correlation with information addiction behavior.*

### 1.2. The Mediation Role of Mindfulness

Mindfulness, characterized by present-moment awareness and non-judgmental acceptance, is not only an individual trait but also one shaped by the familial context. Research indicates that cohesive family environments, rich in emotional support and open communication, foster the development of mindfulness by providing a safe space for individuals to attend to their experiences without excessive criticism or avoidance (Geurtzen et al., 2015; Williams, 2012). Conversely, adverse familial experiences, such as childhood maltreatment or emotionally detached parenting, are associated with lower levels of mindfulness (Arslan, 2017; Yue et al., 2020). Therefore, we posit that family cohesion serves as an antecedent, cultivating a higher capacity for mindfulness.

Regarding the outcome, mindfulness is a well-established protective factor against addictive behaviors, including problematic internet and smartphone use. It enhances self-regulation and reduces experiential avoidance, making individuals less likely to resort to compulsive online activities as a maladaptive coping mechanism (Riley, 2014; Song & Park, 2019). Empirical studies confirm that mindfulness weakens the link between stress and mobile phone addiction (Liu et al., 2018), and that mindfulness-based interventions effectively reduce symptoms of internet addiction (Błachnio et al., 2019). This aligns with the I-PACE model (Brand et al., 2016), which posits that addictive behaviors result from interactions between predisposing variables, affective/cognitive responses, and executive control. Mindfulness can be conceptualized within this framework as a critical cognitive-personal resource that mitigates the maladaptive affective and cognitive responses (e.g., craving and poor impulse control) that lead to compulsive internet use. Building upon the established interplay within family cohesion, mindfulness, and information addiction, we propose our next hypothesis:

**H2.**  *Mindfulness acts as a mediator in the relationship between family cohesion and information addiction.*

### 1.3. The Mediation Role of Anxiety

Family cohesion is a fundamental source of emotional security and coping resources. Robust evidence shows that low family cohesion is a significant risk factor for the development of internalizing symptoms, particularly anxiety (Lucia & Breslau, 2006). In cohesive families, members provide mutual support and effective problem-solving, which buffers against stressors—a function critically important in uncertain contexts like the post-pandemic era. Thus, we argue that lower family cohesion exacerbates anxiety by depriving individuals of a key protective buffer.

Anxiety, in turn, is a potent driver of compulsive information-seeking and internet use. According to the Compensatory Internet Use Theory (CIUT) (Kardefelt-Winther, 2014), individuals may engage in excessive online activities to alleviate or escape from negative emotional states. Anxious individuals often seek certainty, reassurance, or distraction through compulsive information searches and prolonged online engagement (Baumgartner & Hartmann, 2011; te Poel et al., 2016). This aligns with the I-PACE model, which identifies negative affective states like anxiety as core triggering and maintaining variables for specific internet-use disorders. The online environment can serve as an immediate, though ultimately dysfunctional, strategy to regulate anxiety. Hence, we assumed the following hypothesis:

**H3.**  *The relationship between family cohesion and information addiction is mediated by anxiety.*

### 1.4. The Chain Mediating Effect of Mindfulness and Anxiety

The roles of mindfulness and anxiety are not independent but are theoretically and empirically linked. Mindfulness-based theories and interventions explicitly target the reduction of anxiety. By promoting a non-reactive awareness of thoughts and feelings, mindfulness disrupts the cycle of anxious rumination and catastrophic appraisal (Desrosiers et al., 2013; Hofmann et al., 2010; Yang et al., 2025). Meta-analyses confirm that mindfulness training significantly reduces anxiety symptoms (Hofmann & Gómez, 2017; Takhdat et al., 2024; de Oliveira Santana et al., 2024). Therefore, mindfulness is a key antecedent that reduces susceptibility to anxiety.

Integrating this with the pathways outlined above, we propose a coherent chain mediation model grounded in the I-PACE framework. Family cohesion fosters the development of mindfulness (a protective cognitive-personal factor). Higher mindfulness, by enhancing emotion regulation and decreasing cognitive reactivity, leads to lower levels of anxiety (a maladaptive affective response). Finally, reduced anxiety diminishes the need for compensatory internet use, thereby lowering the risk of information addiction. This chain of relationships outlines a more nuanced mechanism through which the family environment ultimately influences digital behavior. According to previous evidence and the I-PACE model, we hypothesize the following:

**H4.**  *Mindfulness and anxiety exhibit a chain mediating role in the relationship between family cohesion and information addiction, with mindfulness negatively associated with anxiety.*

**Figure 1:**
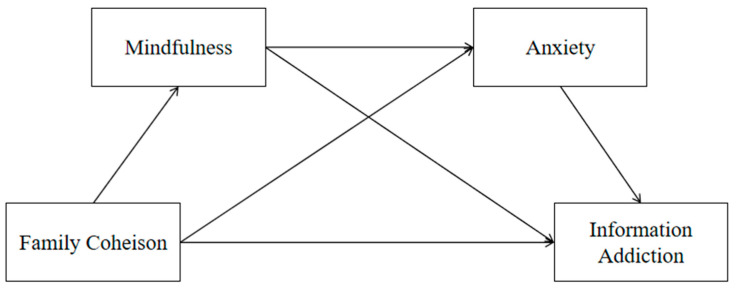
The sequence mediating effect of Information addiction and Family cohesion.

In summary, Family cohesion is the independent variable, Information addiction is the dependent variable, and the mediating variables are Mindfulness and Anxiety. Figure 1 shows the research model of this study. 

## 2. Method

### 2.1. Participants

Utilizing a convenient sampling method, college students from two universities in Tianjin were recruited for a standardized questionnaire through on online platform (https://www.wjx.cn/). Data were collected from August to October 2022. All participants were instructed to provide responses based on their actual experiences and were reassured about the confidentiality of their personal information.

Out of the initial 1043 respondents, 888 valid questionnaires were retained, yielding an effectiveness rate of 85.13%. The sample comprised 486 males (54.7%) and 402 females (45.3%), with a distribution of 389 freshmen, 173 sophomores, 132 juniors, 103 seniors, and 91 graduate students. Ages ranged from 18 to 30, with a mean age of 19.68 years (*SD* = 1.869). 

### 2.2. Measures

#### 2.2.1. Anxiety

The State Trait Anxiety Inventory (STAI) was used to measure the severity of anxiety (Spielberger & Rickman, 1988). For the purpose of this study, we only use the first 20 items, assessing state anxiety in the post-pandemic era (e.g., “I am nervous”). The inventory adopts a 4-point Likert scale, with higher scores indicating increased state anxiety. The Cronbach’s reliability coefficient for the subscale was α = 0.931.

#### 2.2.2. Mindfulness

Mindful Attention Awareness Scale (MAAS) was used to assess the level of mindfulness (Brown & Ryan, 2003). It contains 15 items (e.g., “For me, it’s a piece of cake to concentrate fully”), each inversely presenting the concept of mindfulness on a 6-point scale (1 = almost always; 6 = almost never). A higher score indicates higher levels of mindfulness. The Cronbach’s α in this study was 0.909.

#### 2.2.3. Information Addiction

Information addiction was assessed by the Information addiction Scale that was adapted from Zhou and Young’s research (e.g., “I often spend a lot of time browsing or downloading information online”). The scale encompasses 6 items, rated on a 5-point Likert scale, with “strongly disagree” and “strongly agree” as the extremes. A greater score indicates an increased predisposition towards information addiction. In this study, the scale exhibited a reliability of Cronbach’s α = 0.801.

#### 2.2.4. Family Cohesion 

A translated Chinese version of the Family Adaptability and Cohesion Evaluation Scale (FACESIII) was adapted to measure the level of family cohesion (Olson et al., 1985). Higher scores suggest greater family cohesion and adaptability. Echoing the original, it divides family function into cohesion and adaptability across 20 items (e.g., “Family members seek help from each other”). This study employed the 10 items specifically addressing the cohesion dimension, with a reliability of Cronbach’s α = 0.907.

### 2.3. Procedure and Data Analytic Strategy

The data was input and analyzed using SPSS 26.0, with both descriptive statistical analysis and correlation analysis being performed. Additionally, a chain mediation model was assessed using the Process macro program (Hayes, 2017). A common method bias was analyzed for all variables. Gender and age differences were controlled in the analysis. Specifically, Model 6 was employed to probe the sequence mediating influence of mindfulness and anxiety on the relationship between information addiction and family cohesion. To test the theoretical hypothesis, a statistical analysis estimated a 95% confidence interval (CI) for mediation effects, using a 5000 bootstrap samples (Hayes, 2017).

## 3. Results

### 3.1. Common Method Biases

To address potential common method biases, the Harman single factor test was employed (Podsakoff et al., 2003). The findings revealed the emergence of eight factors with eigenvalues exceeding one. The primary factor accounted for 28.12% of the total variance, which was below the critical threshold of 40%. This suggested that common method bias did not significantly impact the relationships among the study variables.

### 3.2. Descriptive Statistics and Related Analysis of All Variables

Table 1 presented the mean values, standard deviations, and correlation matrix for each variable. Notably, family cohesion, mindfulness, anxiety, and information addiction were positively correlated with each other. Both gender and age exhibited significant correlations with each study variable and will therefore be factored in as control variables in subsequent analyses.

### 3.3. Testing the Sequence Mediating Model

**Figure 2:**
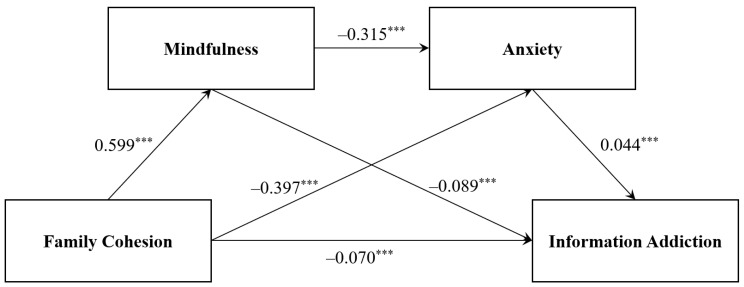
Family Cohesion and Information Addiction in Study. Note. Coefficients are standardized, *** *p* < 0.001.

Table 2 showed the regression result of our model. Family cohesion was found to have a significant positive effect on mindfulness (*β* = 0.599, *t* = 11.499, *p* < 0.001). Meanwhile, mindfulness negatively influenced information addiction (*β* = −0.089, *t* = −7.301, *p* < 0.001) in Model 3. Family cohesion negatively predicted anxiety (*β* = −0.397, *t* = −10.214, *p* < 0.001), while anxiety was a significant positive predictor of information addiction in Model 2 (*β* = 0.045, *t* = 16, *p* < 0.01). The results suggested that mindfulness and anxiety mediated the relationship between family cohesion and information addiction, supporting hypothesis H2: Mindfulness acts as a mediator in the relationship between family cohesion and information addiction, and H3: The relationship between family cohesion and information addiction is mediated by anxiety. Additionally, after adding mediating variables, family cohesion still negatively predicted information addiction (*β* = −0.07, *t* = −3.55, *p* < 0.001), which subsequently supported hypothesis H1: Family cohesion exhibits a negative correlation with information addiction behavior. Mindfulness negatively influenced anxiety (*β* = −0.315, *t* = −13.442, *p* < 0.001). The sequence mediating model was confirmed, supporting the hypothesis H4: mindfulness and anxiety exhibit a chain mediating role in the relationship between family cohesion and information addiction, with mindfulness negatively associated with anxiety (Figure 2).

Total effect, direct effect and indirect effect of mediating model presented in Table 3, verifying effects of three mediated paths. Specifically, family cohesion exhibited a notable predictive power on information collection addiction, boasting a total effect size of −0.149 and a direct effect size of −0.069. The combined mediating effect of mindfulness and anxiety respectively stood at −0.080, and accounted for 53.691% of the total effect, comprised by three paths (P1: Family Cohesion → Mindfulness → Information addiction; P2: Family Cohesion → Anxiety → Information addiction; P3: Family Cohesion → Mindfulness → Anxiety → Information addiction). The mediating effect of mindfulness had a magnitude of −0.054 and constitutes 36.241% of the total effect, while the mediating effect of anxiety quantified at −0.018, making up 12.081% of the total effect. The chain mediating effect showed the weakest mediating effect, accounting for 5.370% of the total effect. Each of the pathways possessed 95% confidence intervals that did not include 0 through a bootstrap analysis, which highlighted the significance of the effects across all pathways.

## 4. Discussion

This study aims to explore the relationship between digital information dependency behaviors and family cohesion among university students in environments characterized by uncertainty, as well as the underlying mechanisms involved. The findings indicate a significant negative correlation between family cohesion and the degree of information addiction. The result suggested that family cohesion was negatively correlated with information addiction. Individuals residing within families characterized by poor cohesion are susceptible to information addiction, aligning with previous research on internet addiction and family dynamics (Chung et al., 2019). Family cohesion embodies the emotional bonds that connect family members (Olson et al., 2019). Drawing from the conservation of resource theory (Hobfoll et al., 1990), individuals with a high-cohesion family can receive more emotional support (Farrell & Barnes, 1993). This serves as a safeguard against the onset of negative emotional states, which diminishes the inclination to resort to the internet as a compensatory resource, consequently decreasing tendencies toward information addiction (Salimi et al., 2016; W. Li et al., 2014). The results showed that focusing on strategies to enhance family cohesion can help alleviate internet addiction issues, particularly among younger generations. 

Mindfulness mediated the association between family cohesion and information addiction. Strong family cohesion enhances individual mindfulness, thereby reducing vulnerabilities to information addiction, aligning with findings from previous research (Arslan, 2017; Chuan et al., 2022). A better family environment can cultivate mindfulness in the individual (S. Zhou et al., 2025). Also, previous research confirmed that mindfulness is effective in reducing stress, regulating emotions, and mitigating negative emotional states (Finkelstein-Fox et al., 2018). The protective scope of mindfulness extends to mitigating addictive behavioral tendencies, particularly those related to mobile and internet addiction (D. Li et al., 2023). Therefore, innovative interventions targeting internet addiction can be designed to improve individuals’ levels of mindfulness. 

The result employed a mediated model of anxiety and gained a deeper understanding of the relationship. Stronger family cohesion can serve as a protective factor against anxiety, which may induce further internet addiction problems, corresponding to the previous research (Adams et al., 2019; Liu et al., 2020). The context of the post-pandemic era has given rise to widespread emotional disorders, including anxiety, depression, heightened stress, and disrupted sleep patterns. During this period, weaker family cohesion associated with worse stress consequences among college students (Zeng et al., 2021). Low family cohesion and connection are related with a higher prevalence of anxiety symptoms (Anyan & Hjemdal, 2018; Watkins et al., 2022). Furthermore, elevated levels of anxiety are correlated with an increase in information addiction behavior (te Poel et al., 2016). Predominantly, individuals with elevated anxiety levels tend to interpret health-related information through a more catastrophic cognitive lens, resulting in heightened health anxieties—a phenomenon termed “cyberchondria” (Barke et al., 2016; Starcevic & Berle, 2013; Zheng et al., 2020). Anxiety triggers heightened information-seeking behavior due to individuals’ need for information in response to potential threats, thereby alleviating the anxiety induced by crisis (Qin et al., 2021; Wang et al., 2021). These findings indicate the underlying mechanism of how a predisposition, such as family cohesion, influences internet addiction by elevating anxiety levels.

The study identified a chain mediating model linking family cohesion and information addiction through sequential mediation by mindfulness and anxiety. A well-functioning family can improve psychological well-being through different social circumstances (Vandeleur et al., 2009). Individuals who live in a less-connected family setting are more likely to develop psychological and behavioral problems, such as addiction (Tafa & Baiocco, 2009). 

A positive parent-child relationship and emotional support are beneficial for mindfulness, emotional regulation, and social-emotional development at all stages of development (Lunkenheimer et al., 2007; Siegel, 2012; Morelen & Suveg, 2012; Skoranski et al., 2019). Moreover, evidence showed that mindfulness and attachment with parents share similar neurophysiology (Brown et al., 2015). Individuals who have stronger child–parent relationships tend to exhibit higher levels of mindfulness, consequently rendering them less susceptible to anxiety-related issues. Engaging in excessive information consumption serves as a coping mechanism for managing anxiety and other psychological challenges in the post-pandemic era, particularly in the absence of strong family cohesion and emotional support, thus reinforcing the theoretical framework.

By focusing on information addiction during the post-pandemic period, this study underscores the protective role of family cohesion and clarifies the psychological pathways involved. The mediating effects of mindfulness and anxiety emphasize the need for interventions that address both emotional regulation and coping strategies. Overall, this research extends theoretical models of digital addiction and family psychology, while supporting the development of multimodal prevention and treatment strategies that enhance familial and individual resilience against problematic information use.

However, our study is not devoid of limitations. Primarily, our exploration was confined to the relationship between family cohesion and information addiction, neglecting a detailed examination of the specific symptoms and severity of information addiction. Additionally, we did not investigate the contextual specificity of information in the post-pandemic era, whether it is health-related or otherwise. Future studies should aim to explore the nuances of different types of information and the associated symptoms and intensities of information addiction. Methodologically, reliance on self-report measures, such as questionnaires, may cause response bias. Also, the application of a cross-sectional design was insufficient for establishing a causal interpretation. Furthermore, participants primarily consisted of college students, highlighting the need for future studies to encompass a broader age range, possibly through longitudinal methodologies. Looking ahead to future research trajectories, it is imperative to delve into the multifaceted mechanisms underlying information addiction, encompassing its etiological foundations and its subsequent impacts on overall well-being.

## 5. Conclusions

The study explored a sub-dimension of internet addiction, named information addiction, during a public health emergency to better understand the profound impact of internet technology during crisis. The result found that family cohesion is negatively correlated with information addiction, with mindfulness and anxiety serving as mediators. Individuals with lower family cohesion tend to exhibit lower levels of mindfulness and higher levels of anxiety, consequently fostering compulsive information-seeking behavior. The result also employed a chain mediating model between family cohesion and information addiction. Higher family cohesion can predict a greater level of mindfulness, which in turn negatively influences anxiety symptoms, leading to a reduction of information addiction behavior. This research reaffirms the significant impact of constructs such as family cohesion and information addiction on individual mental well-being during periods of profound social change. It underscores the increased need for a heightened focus on mental well-being and coping strategies during epidemics.

## Figures and Tables

**Table 1:** Descriptive statistical results and correlation matrix between variables.

	M (SD)	Skewness	Kurtosis	1	2	3	4
1 Family Cohesion	37.36 (7.855)	−0.528	0.219	-			
2 Mindfulness	62.71 (13.193)	−0.402	0.311	0.363 **	-		
3 Anxiety	38.84 (10.36)	0.265	−0.126	−0.441 **	−0.492 **	-	
4 Information Addiction	15.89 (4.427)	0.371	−0.196	−0.263 **	−0.344 **	0.294 **	-

Note. ** *p* < 0.01.

**Table 2:** Regression Analysis of Family Cohesion, Mindfulness, Anxiety, and Information Addiction.

Variable	Mindfulness			Anxiety			Information Addiction		
	*B*	*SE*	*t*	*B*	*SE*	*t*	*B*	*SE*	*t*
Age	0.5	0.213	2.283 *	0.306	0.153	1.984 *	0.318	0.073	4.348 ***
Sex	3.132	0.824	3.802 ***	2.062	0.578	3.568 ***	0.46	0.278	1.656
Cohesion	0.599	0.052	11.499 ***	−0.397	0.039	−10.214 ***	−0.07	0.02	−3.550 ***
Mindfulness				−0.315	0.023	−13.467 ***	−0.089	0.012	−7.285 ***
Anxiety							0.044	0.016	2.745 **
*R* ^2^	0.153			0.334			0.172		
*F*	53.119 ***			110.764 ***			36.706 ***		

Note. * *p* < 0.05, ** *p* < 0.01, *** *p* < 0.001.

**Table 3:** Decomposing the Mediating Effect of Mindfulness and Anxiety.

	Effect	BootSE	BootLLCI	BootULCI	Relative Mediating Effect
TOTAL	−0.080	0.012	−0.105	−0.056	53.691%
Ind 1	−0.054	0.009	−0.073	−0.037	36.241%
Ind 2	−0.018	0.007	−0.031	−0.005	12.081%
Ind 3	−0.008	0.003	−0.015	−0.003	5.370%

## Data Availability

The datasets generated and analyzed during this study are not publicly available as they constitute the intellectual property and proprietary assets of the research team. Requests for access to these data can be directed to the corresponding author and will be considered on a case-by-case basis, subject to compliance with applicable privacy and confidentiality agreements.
